# Endemic Chromoblastomycosis Caused Predominantly by *Fonsecaea nubica*, Madagascar^1^

**DOI:** 10.3201/eid2606.191498

**Published:** 2020-06

**Authors:** Tahinamandranto Rasamoelina, Danièle Maubon, Malalaniaina Andrianarison, Irina Ranaivo, Fandresena Sendrasoa, Njary Rakotozandrindrainy, Fetra A. Rakotomalala, Sébastien Bailly, Benja Rakotonirina, Abel Andriantsimahavandy, Fahafahantsoa Rakato Rabenja, Mala R. Andrianarivelo, Muriel Cornet, Lala S. Ramarozatovo

**Affiliations:** Université d’Antananarivo, Antananarivo, Madagascar (T. Rasamoelina, N. Rakotozandrindrainy, F.A. Rakotomalala, B. Rakotonirina, A. Andriantsimahavandy, M.Rakato Andrianarivelo);; Université Grenoble Alpes, Grenoble, France (D. Maubon, S. Bailly, M. Cornet);; Hôpital Universitaire Joseph Raseta Befelatanana, Antananarivo (M. Andrianarison, I. Ranaivo, F. Sendrasoa, F.R. Rabenja, L.S. Ramarozatovo);; Centre Hospitalier Universitaire de Befelatanana, Antananarivo (L.S. Ramarozatovo)

**Keywords:** chromoblastomycosis, dermatomycosis, fungi, fungal infections, epidemiology, prevalence, molecular diagnosis, clinical presentation, clinical outcome, Fonsecaea nubica, Fonseca pedrosoi, Madagascar

## Abstract

Chromoblastomycosis is an implantation fungal infection. Twenty years ago, Madagascar was recognized as the leading focus of this disease. We recruited patients in Madagascar who had chronic subcutaneous lesions suggestive of dermatomycosis during March 2013–June 2017. Chromoblastomycosis was diagnosed in 50 (33.8%) of 148 patients. The highest prevalence was in northeastern (1.47 cases/100,000 persons) and southern (0.8 cases/100,000 persons) Madagascar. Patients with chromoblastomycosis were older (47.9 years) than those without (37.5 years) (p = 0.0005). Chromoblastomycosis was 3 times more likely to consist of leg lesions (p = 0.003). Molecular analysis identified *Fonsecaea nubica* in 23 cases and *Cladophialophora carrionii* in 7 cases. Of 27 patients who underwent follow-up testing, none were completely cured. We highlight the persistence of a high level of chromoblastomycosis endemicity, which was even greater at some locations than 20 years ago. We used molecular tools to identify the *Fonsecaea* sp. strains isolated from patients as *F. nubica*.

Chromoblastomycosis is a chronic, implantation, fungal disease caused by melanized fungi from a variety of genera of the order Chaetothyriales. This disease is included in a group of melanized infections and easily identifiable by verrucous lesions that eventually lead to cauliflower-like eruptions on the skin. Infection is acquired traumatically by implantation of infected plant material from thorns or wood splinters or by soil contamination of an existing wound (*1*,*2*). The causative agents are mainly *Fonsecaea* spp., *Cladophialophora* spp., and *Rhinocladiella* spp. However, rare cases caused by other genera, such as *Phialophora* spp. or *Exophiala* spp., have been reported (*1*,*3*). As is the case for other implantation mycoses, chromoblastomycosis lesions are located mainly on the lower limbs, particularly on the dorsal face of the feet, ankles, and legs (*1*,*4**–**6*).

Infection is caused by a lack of protective clothing or shoes for persons working in rural areas in which spiny plants are common. Chromoblastomycosis is linked to poverty and is highly prevalent in low-income resource countries. It was the first fungal infection to be recognized as a neglected tropical disease (along with mycetoma, which is not exclusively of fungal origin) (*7*).

The clinical manifestation of chromoblastomycosis is polymorphous but is dominated by verrucous and tumoral lesions resembling cauliflower. No clinical particularity associated with the fungal species or genera has been described (*1**,**4**,**5**,**7*). Infection begins with development of muriform cells in the skin, provoking a granulomatous immune response. Muriform cells are specific to chromoblastomycosis and described as large brown, thick-walled, compartmented cells. They are also found in the infected plants assumed to be the source of human contamination.

Albeit belonging to the same order, the species found in these plants and the soil are different from the pathogenic ones (*4**,**5**,**7**,**8*). Infection involves >1 nodules that develop into verrucous, hyperkeratotic, or papillomatous lesions or plaques. The lesions progress slowly, over a period of 2–20 years and become highly disabling because of development of elephantiasis-type edema or superinfections. Itching and scratching favor dissemination (*1*). The usual diagnosis relies on detection of the muriform cells in superficial samples, which is sufficient to confirm chromoblastomycosis. However, culture, yielding black fungi, is required to identify the causative agent at the species level by morphologic and molecular analyses (*1**,**5*).

Chromoblastomycosis predominates in tropical and subtropical regions, and most reported cases are from Latin America (Brazil, Mexico, and Venezuela), the Caribbean (Dominican Republic and Cuba), Africa, Asia (India, Japan, and southern China) and Australia (*1*). In Madagascar, studies conducted by Institut Pasteur during 1955–1994 provided an inventory of the number of cases of chromoblastomycosis and identified this country as the leading focus of chromoblastomycosis worldwide. The mean annual incidence was estimated to be ≈1 cases/200,000 persons during this period. The most commonly isolated agents were *Fonsecaea pedrosoi* in the humid tropical areas and *Cladophialophora carrionii* in the semiarid zones of the southern Madagascar (*9**–**11*).

Since 2013, we have established a cross-sectional study to document the current epidemiology of implantation mycoses in Madagascar, including chromoblastomycosis (*12*). Clinical diagnosis and fungal identification were confirmed by using molecular biology methods. We describe the current prevalence and clinical manifestation of chromoblastomycosis in Madagascar and patient outcomes. We also report the species-level identification, genetic relatedness, and antifungal susceptibility of clinical isolates.

## Materials and Methods

### Study Design and Patient Recruitment

**Figure 1:**
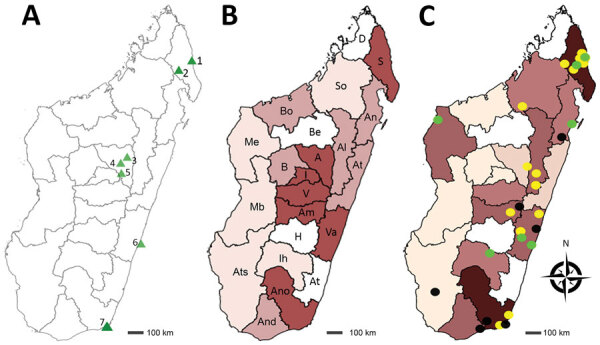
Recruitment of patients for study of chromoblastomycosis and prevalence by region, Madagascar, March 2013–June 2017. A) Recruitment sites (green triangles). Region of Sava: 1) Centre Hospitalier de Référence Régionale, Sambava District; 2) Centre Hospitalier de District and Hôpital Adventiste, Andapa District, Analamanga Region; 3) Centre de Santé de Base Alakamisy-Anjozorobe, Anjozorobe District; 4) Centre Hospitalier Universitaire Joseph Ravoahangy Befelatanana, Antananarivo District; 5) Centre de Santé de Base, Andramasina District, Vatovavy Fitovinany Region; 6) Fondation Médicale Ampasimanjeva, Manakara District; Anosy Region; 7) Centre Médical Tolagnaro, Centre Hospitalier de Référence Régionale Tolagnaro and Hôpital Luthérien Manambaro, Tolagnaro District. B) Geographic origin of patients recruited. Regions from north to south: D, Diana; S, Sava; I, Itasy; A, Analamanga; V, Vakinankaratra; B, Bongolava; So, Sofia; Bo, Boeny; Be, Betsiboka; Me, Melaky; Al, Alaotra-Mangoro; At, Atsinanana, An, Analanjirofo; Am, Amoron’I Mania; H, Haute Matsiatra; Va, Vatovavy-Fitovinany; Ato, Atsimo-Atsinanana; Ih, Ihorombe; Mb, Menabe; Ats, Atsimo Andrefana; And, Androy; Ano, Anôsy. No. patients recruited; dark purple, >6; medium purple, 3–5; light purple, <3; white, missing (none). C) Geographic distribution of chromoblastomycosis cases and causative fungal agents. Prevalence is no. cases/100,000 persons: dark purple, >0.5; medium purple, 0.1–0.5; light purple, <0.1; white, missing (none). Causative agent distribution: yellow dots, *Fonsecaea nubica*; black dots, *Cladophialophora carrionii*; green dots, *Fonsecaea* sp.

We conducted a cross-sectional study as described (*12*). We recruited patients with clinically suspected chromoblastomycosis or another chronic dermatomycosis during March 2013–June 2017 at the Dermatology Department of the Joseph Raseta Befelatanana University Hospital in Antananarivo or during advanced consultation campaigns in districts (Figure 1, panel A). A clinical and demographic information form was completed for each participant. This study was approved by the Ethics Committee for Biomedical Research of the Ministry of Public Health of Madagascar (authorization no. 66-MSANP/CE).

### Case Definition

We provide clinical, mycological, histological, severity and prognostic criteria used for classifying cases in this study (Table 1). Cases were identified after a monthly consultation between the clinicians of the Department of Dermatology-Rheumatology of Joseph Ravoahangy Befelatanana University Hospital Center in Antananarivo and the teams of mycologists from the Charles Mérieux Infectiology Center and Université Grenoble Alpes.

**Table 1:** Classification criteria for cases of endemic chromoblastomycosis caused predominantly by *Fonsecaea nubica*, Madagascar*

Criteria	Description
Clinical	
Major	1) Nodular: moderately elevated, fairly soft, dull to pink violaceous growth; surface is smooth, verrucous, or scaly.
	2) Verrucous: hyperkeratosis is the outstanding feature; warty dry lesions; frequently encountered along the border of the foot.
	3) Tumorous: tumor-like masses, prominent, papillomatous, sometimes lobulated; cauliflower like; surface is partly or entirely covered with epidermal debris and crusts; more exuberant on lower extremities.
	4) Cicatricial: nonelevated lesions that enlarge by peripheral extension with atrophic scarring, while healing takes place at the center; might expand centrifugally, usually with an annular, arciform, or serpiginous outline; tends to cover extensive areas of the body.
	5) Plaque: least common type; slightly elevated with areas of infiltration of various sizes and shapes; red to violet color; a scaly surface, sometimes showing marked lines of cleavage; generally found on the higher portions of the limbs, shoulders, and buttocks.
	6) Mixed form: association of the 5 basic types of lesions; usually observed in patients showing severe and advanced stages of the disease.
	7) Clinical form on the face: erythematosquamous cup, central plate, atrophic, cicatricial, retractile, papular on the face, edema on the lips.
Minor	Pseudovacuolar and eczematous types in patients with a short time of evolution (<3 mo)
Mycological and histological	
Major	1) Muriform cells found by direct microscopic examination or histological analysis.
	2) Molecular evidence of *Fonsecaea* spp., *Cladophialophora carrionii*, or *Rhinocladiella aquaspersa* by PCR with specific primers or internal transcribed spacer, BT2, or TF1 sequencing directly from clinical samples or a positive fungal culture of a melanized fungus morphologically reminiscent of *Fonsecaea* spp., *C. carrionii*, or *R. aquaspersa*.
	3) Nonambiguous identification (score >2) of *Fonsecaea* spp., *C. carrionii*, or *R. aquaspersa* by MALDI-TOF MS with a validated main spectra profile.
Minor	Positive fungal culture of a melanized fungus morphologically reminiscent of *Fonsecaea* sp., *C. carrionii*, or *R. aquaspersa* from a clinical sample without molecular confirmation or ambiguous identification (score <2) of *Fonsecaea* spp., *C. carrionii*, or *R. aquaspersa* by MALDI-TOF MS with a home-made validated main spectra profile.
Classification	
Confirmed	>1 of the major clinical criteria and >1 of the major mycological criteria or 1 minor clinical criterion and >1 of the major mycological criteria
Probable	>1 of the major clinical criteria and 1 minor mycological or histological criterion and a complete or partial response to antifungal therapy
Possible	>1 of the major clinical criteria without any (major or minor) mycological or histological criteria or >1 of the minor clinical criteria without any (major or minor) mycological or histological criteria and a complete or partial response to antifungal therapy
Severity	
Mild	Solitary plaque or nodule <5 cm in diameter
Moderate	Solitary or multiple lesions as nodular, verrucous, or plaque types existing alone or in combination, covering 1 or 2 adjacent cutaneous regions and measuring <15 cm in diameter
Severe	Any type of lesion alone or in combination covering extensive cutaneous regions whether adjacent or nonadjacent
Clinical response during antifungal therapy	
Major	Resolution of lesions with no relapse after 6 mo of follow-up. Reduction in the thickness/induration of lesions by 75% or reduction of the surface area affected by palpable lesions by 75%
Minor	Resolution of all cutaneous symptoms (i.e., pruritus) referable to the lesions and some objective improvement of lesions, less than a major response
Failure	Minor improvement or no change, worsening of lesions on therapy

*Adapted from Queiroz-Telles et al. (*1*). MALDI-TOF MS, matrix-assisted laser desorption/ionization time-of-flight mass spectrometry.

### Clinical Samples

We obtained consent from patients and collected specimens consisting of biopsy material or flakes of skin. We then sent samples to the laboratory of the Charles Mérieux Infectiology Center of Antananaivo, where they were processed immediately or after 24–48 hours of storage at 2°C–8°C.

### Mycological Analysis

#### Cultures

We performed direct microscopic examination of clinical specimens with and without Chlorazol Black staining to detect muriform cells, which are typical of chromoblastomycosis (*2*). We then used samples to inoculate Sabouraud medium supplemented with chloramphenicol, on which samples were incubated at 30°C for 2–3 weeks. For positive cultures, we morphologically identified fungal isolates, extracted DNA, and froze the culture at −80°C.

### Molecular Analysis

We used the QIAamp DNA Blood Mini Kit (QIAGEN, https://www.qiagen.com) for DNA purification from clinical samples and fungal colonies. We performed PCR amplification in 2 steps. In the first step, we used 2 panfungal PCRs targeting internal transcribed spacer (ITS) regions with primers ITS1/ITS4 and D1D2 regions with primers NL-1/NL-4 and NL-3/NL-4 (*13**–**15*). In the second step, we used a *C. carrionii*–specific PCR, primers Ccar-F 5′-ATCGCTGCGAAGCGTCTCG-3′ and Ccar-R 5′-ACCGTCCAACACCAAGCACAGG-3′, and specific *Fonsecaea* sp. and PCR primers that have been described (*16*). We sequenced panfungal PCR products by LGC Genomics GmbH, https://www.nucleics.com) by using the same primers as for amplification.

We aligned the sequences obtained for panfungal PCR with reference sequences in the International Society of Human and Animal Mycology Barcoding Database (http://its.mycologylab.org) for the ITS region and the National Center for Biotechnology Information (NCBI; https://www.ncbi.nlm.nih.gov) database for the D1D2 and ITS regions (*17*). We constructed a phylogenetic tree by using MEGA7 software (https://www.megasoftware.net) according to the protocol of Barry G. Hall (Bellingham Research Institute, Bellingham, WA, USA), based on the maximum-likelihood method.

### Matrix-Assisted Laser Desorption/Ionization Time-of-Flight Mass Spectrometry Analysis

In-house main spectrum profiles (MSPs) were created on the Microflex Mass Spectrometer (Bruker Daltonics, https://www.bruker.com) according to the MALDI Biotyper MSP Creation version 1.1 protocol for reference strains of *C. carrionii*, *F. nubica*, *F. pedrosoi*, and *F. monophora* (1 of each) and 7 isolates formally identified by ITS DNA sequencing (Appendix Table). Isolates were cultured under 3 conditions: in Sabouraud–chloramphenicol agar for 4–7 days at 30°C, in liquid Sabouraud medium for 2–4 days at 25°C–30°C with shaking, and on solid peptone dextrose agar for 4–5 days at 30°C. We used an external validation of the new library performed with clinical isolates obtained during the study but not used to create the MSPs. We made a rapid identification by using a direct deposition method in accordance with MALDI Biotyper In Vitro Diagnostic Protocol Version 1.6 (Bruker Daltonics). We compared spectra obtained with Bruker Taxonomy (7,815 entries), Bruker Filamentous Fungi (364 MSP), NIH mold (365 profiles) (*18*), and MSP-chromoblastomycosis in-house databases and generated identification scores with the following quality criteria: score >2, species-level identification; score <1.7–<2, genus-level identification; score <1.7, no identification.

### Susceptibility to Antifungal Drugs

The M38-A2 protocol of the Clinical and Laboratory Standard Institute for filamentous fungi was used on mycelial strains after subculture at 30°C to determine the MICs for antifungal agents (*19*). The following agents were tested at the concentrations indicated: posaconazole and isavuconazole, 0.016–8 μg/mL; amphotericin B and itraconazole, 0.006–32 μg/mL; and terbinafine, 0.008–4 μg/mL. MICs were determined after 120 h of culture at 30°C. We used a 100% inhibition endpoint for all drugs except for terbinafine, for which the endpoint was 80%.

### Statistical Analysis

We compared chromoblastomycosis cases and other nonchromoblastomycosis cases by using χ^2^ or Fisher exact tests for qualitative variables and Student *t*-tests for quantitative variables. Because this infection is chronic, we calculated prevalence by dividing the total number of cases at the end of the study period in June 2017 by the number of persons in the area concerned. We calculated the number of persons at the end of the period from the most recent figures available in 2013 from the official website of the National Institute of Statistics of Madagascar (*20*) and adjusted for the subsequent years with a growth rate of 2.7% per year (World Bank estimates of demographic growth in Madagascar). We analyzed data and generated maps by using Epi Info version 7.2.2.1(*21*) and R Studio version 1.0.153 (*22*).

## Results

### Demographic and Clinical Characteristics of Patients

During March 2013–June 2017, we included 148 patients with chronic cutaneous or subcutaneous lesions in the study. The mean (SD) age of the patients was 41 (18.8) years; 111 (75.0%) were males. The largest number of patients (n = 118, 79.7%) was enrolled at Joseph Raseta Befelatanana University Hospital, the permanent recruitment center (Figure 1, panel A). An analysis of the geographic origin of the patients showed that most (n = 90, 60.8%) patients came from the highlands, followed by the regions in the northeast (n = 23, 15.5%), east and southeast (n = 16, 10.8%), south and southwest (n = 13, 8.8%), and west (n = 6, 4.1%) (Figure 1, panel B). A comparison of the full years of recruitment (2014, 2015, and 2016) showed that the number of patients was higher in 2015 (n = 47, 31.8%) and 2016 (n = 36, 24.3%) than in 2014 (n = 28, 18.9%), but this difference was not significant (p = 0.47).

The largest proportion of the patients worked in agriculture (n = 76, 51.3%), followed by the service sector (n = 31, 21%), students (n = 20, 13.5%), craftsmen (n = 14, 9.5%), and the unemployed (n = 7, 4.7%). Lesions were located principally on the legs (62.8%) and arms (28.3%).

### Patients with Chromoblastomycosis

At the first consultation, 58 of 148 patients had clinically suspected chromoblastomycosis. A diagnosis of chromoblastomycosis was made for 50 (33.8%) patients: confirmed for 41 (27.7%), probable for 3 (2.0%), and possible for 6 (4.0%). The frequency of chromoblastomycosis remained stable during 2013–2017 (21.4%–47.2%; p = 0.12 (Table 2) During 2014–2016, the mean (SD) number of annual chromoblastomycosis cases was 12 (5.5).

**Table 2:** Characteristics of chromoblastomycosis cases in patients with chronic cutaneous and subcutaneous lesions, Madagascar, March 2013–June 2017*

Characteristic	Chromoblastomycosis				Other,‡ n = 98	p value
	Severe,† n = 27	Moderate,† n = 21	NA, n = 2	All, n = 50		
Period of recruitment						
2013, starting March 1	NS	NS	NS	6 (12.0)	10 (10.2)	0.12
2014	NS	NS	NS	6 (12.0)	22 (22.4)	NS
2015	NS	NS	NS	12 (24.0)	35 (35.7)	NS
2016	NS	NS	NS	17 (34.0)	19 (19.5)	NS
2017, through May 31	NS	NS	NS	9 (18.0)	12 (12.2)	NS
Mean age, y, (SD)	NS	NS	NS	47.9 (15.7)	37.5 (19.4)	0.0005
Age range, y						
3–17	NS	NS	NS	1 (2.0)	16 (16.3)	0.001
18–32	NS	NS	NS	5 (10.0)	26 (26.5)	NS
33–47	NS	NS	NS	21 (42.0)	26 (26.5)	NS
48–62	NS	NS	NS	10 (20.0)	19 (19.4)	NS
63–80	NS	NS	NS	13 (26.0)	11 (11.2)	NS
Sex						
M	NS	NS	NS	46 (92.0)	65 (66.3)	0.001
F	NS	NS	NS	4 (8.0)	33 (33.7)	NS
Occupation						NS
Farmer	17 (63.0)	12 (57.1)	2 (100.0)	31 (62.0)	45 (45.9)	0.006
Services sector	7 (25.9)	7 (33.3)	0	14 (28.0)	17 (17.4)	NS
Student	1 (3.7)	1 (4.8)	0	2 (4.0)	18 (18.4)	NS
Merchant-artisan	1 (3.7)	0	0	1 (2.0)	13 (13.3)	NS
Unemployed	1 (3.7)	1 (4.8)	0	2 (4.0)	5 (5.1)	NS
Anatomic location						
Lower limb	21 (77.8)	17 (81.0)	2 (100.0)	40 (80.0)	53 (54.1)	0.005
Upper limb	3 (11.1)	3 (14.3)	0	6 (12.0)	36 (37.7)	NS
Other§	3 (11.1)	1 (4.7)	0	4 (8.0)	9 (9.2)	NS
Duration of the lesion, y						
<1	2 (7.4)	1 (4.8)	0	3 (6.0)	56 (57.1)	<0.0001
1–2	1 (3.7)	1 (4.8)	0	2 (4.0)	14 (14.3)	NS
>2	24 (88.9)	19 (90.4)	2 (100.0)	45 (90.0)	28 (28.6)	NS
Clinical form						
Nodular	1 (3.7)	3 (14.3)	0	4 (8.0)	NS	NS
Verrucous	1(3.7)	4 (19.0)	0	5 (10.0)	NS	NS
Tumorous	5 (18.5)	0	0	5 (10.0)	NS	NS
Cicatricial	3 (11.1)	1 (4.8)	0	4 (8.0)	NS	NS
Plaque	3 (11.1)	7 (33.3)	0	10 (20.0)	NS	NS
Mixed	8 (29.6)	3 (14.3)	0	11 (22.0)	NS	NS
Mixed on the face	2 (7.4)	0	0	2 (4.0)	NS	NS
Modified by previous therapy	4 (14.8)	3 (14.3)	0	7 (14.0)	NS	NS
NA	0	0	2 (100.0)	2 (4.0)	NS	NS

*Values are no. (%) unless otherwise indicated. NA, not available; NS, not specified. †For definitions, see Table 1. ‡Sporotrichosis (n = 63) (*12*); mycetoma (n = 4); carcinoma, eczema, and cutanesous tuberculosis (n = 2); carpitis, erysipela, fibrosarcoma, cutaneous lymphoma, porokeratosis of Mibelli, prurigo nodularis, pyoderma, and papillomavirus (n = 1); not specified (n = 17). §Head and neck, thorax.

Patients who had chromoblastomycosis were significantly older (47.9 years) than those without chromoblastomycosis (37.5 years) (p = 0.0005). Analysis by age group showed that this trend was linked to a higher frequency of chromoblastomycosis in persons 33–48 years of age (44.0%) and 63–80 years of age (54.1%) (p = 0.001). The risk for having chromoblastomycosis was almost 5 times higher after the age of 33 years (odds ratio 5.44, 95% CI 2.04–17.10; p = 0.0001).

Male predominance was more marked in patients with chromoblastomycosis (92.0%) than in the other recruited patients (66.0%) (p = 0.001) (Table 2). Chromoblastomycosis patients were predominantly farmers and employees of the service sector (Table 2). The risk for chromoblastomycosis tended to be higher among farmers than among persons with other professions grouped together (OR 1.92, 95% CI 0.95–3.85; p = 0.09).

Location of lesions differed between chromoblastomycosis patients and other patients. Overall, 80.0% of patients with chromoblastomycosis had leg lesions, compared with 54.1% of patients without chromoblastomycosis (p = 0.005) (Table 2). A diagnosis of chromoblastomycosis was 3 times more likely than any other diagnoses for leg lesions (OR 3.36, 95% CI 1.45–8.4; p = 0.003).

**Figure 2:**
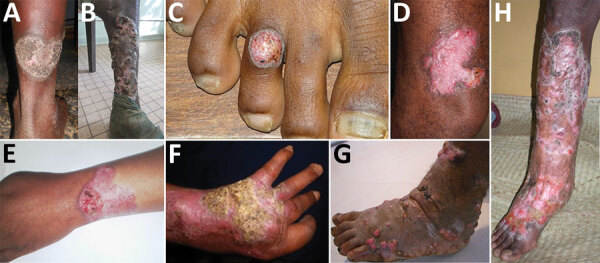
Clinical forms of chromoblastomycosis caused by *Fonsecaea* sp., Madagascar. A) Plaque; B) mixed: tumorous and cicatricial; C) nodular; D) raised plaque; E) plaque; F) cicatricial; G) tumorous caused by *Cladophialophora carrionii*; H) mixed: cicatricial and modified by previous therapy.

Mixed and tumorous lesions were the most frequent forms in chromoblastomycosis cases (Table 2; Figure 2). Lesions were mostly severe (54%) and moderate (42%). All tumorous forms were severe. Chromoblastomycosis lesions had been present for >1–2 years in 90% of patients. The longest duration of lesion presence was 36 years. All but the verrucous forms were seen after 2 years of evolution (Table 2).

### Prevalence and Geographic Distribution

We determined the geographic origin of patients with chromoblastomycosis, corresponding to the presumed origin of contamination. Most (84.0%) chromoblastomycosis patients originated from peripheral regions, such as the northern (40%), eastern and southeastern (22%), and southern and southwestern (20%) areas. We observed only 8 (16.0%) cases in the central highlands. In June 2017, the prevalence of chromoblastomycosis was highest (1.47 cases/100,000 persons) in the Sava region in northeastern Madagascar, followed by the Anosy region in south Madagascar (0.80 cases/100,000 persons) (Figure 1, panel C; Table 3).

**Table 3:** Prevalence of chromoblastomycosis, Madagascar, March 2013–June 2017

Region	No. persons*	No. cases	Prevalence
North and central north	3,629,908	20	0.55
Analanjirofo	1,151,536	2	0.17
Sava	1,091,102	16	1.47
Sofia	1,387,270	2	0.14
Highlands	7,850,950	8	0.10
Analamanga	3,725,377	3	0.08
Amoron’i Mania	795,434	3	0.38
Itasy and Bongolava	1,324,044	0	0
Vakinankaratra	2,006,095	2	0.10
West	1,870,459	1	0.05
Boeny and Menabe	1,548,299	0	0.00
Melaky	322,160	1	0.31
East and South East	4,131,928	11	0.27
Alaotra Mangoro	1,142,612	4	0.35
Atsinanana	1,413,572	1	0.07
Vatovavy Fitovinany	1,575744	6	0.38
South and South West	3,376,074	10	0.30
Androy	816,466	3	0.37
Anosy	747,352	6	0.80
Atsimo Andrefana	1,464,830	0	0
Ihorombe	347,427	1	0.29

*Determined at the end of the study period and calculated from the most recent figures available in 2013 and adjusted for subsequent years with a growth rate of 2.7%/year (World Bank estimates of demographic growth in Madagascar). †No. cases/100,000 persons.

### Mycological Results

We collected 192 samples (151 biopsy specimens, 23 skin flake samples, and 18 pus samples) from 148 patients. For chromoblastomycosis patients, we analyzed 58 samples (47 biopsy specimens, 7 skin flake samples, and 4 pus samples). We compiled results of mycological investigations, including molecular analyses of samples from chromoblastomycosis patients (Appendix Table). Direct examination showed muriform cells in 33 (56.9%) samples, 145 (90.6%) biopsy specimens, and 13 (57.1%) skin flake samples (p = 0.11).

#### Culture

We obtained 172 cultures, including 41 from samples of 50 chromoblastomycosis patients. Overall, 26 (63.4%) cultures had macroscopic morphological features consistent with *Fonsecaea* sp. and *C. carrionii*, and 2 (4.8%) cultures had microscopic morphological features consistent with *Fonsecaea* sp. and *C. carrionii*.

#### Molecular Analysis

The ITS panfungal PCR had lower sensitivity for clinical specimens than D1D2 PCR (21.6% vs. 91.9%; p<0.0001). The performances of the 2 panfungal PCR tests were relatively similar with cultures (75.0% vs. 92.5%; p *=* 0.07). Concerning the sensitivity of specific PCRs for clinical specimens, the PCR for *C. carrionii* was unable to confirm identification for any of the specimens, whereas the PCR for *Fonsecaea* spp. established a diagnosis in 16 (55.3%) of 30 cases caused by this genus (Appendix Table).

**Figure 3:**
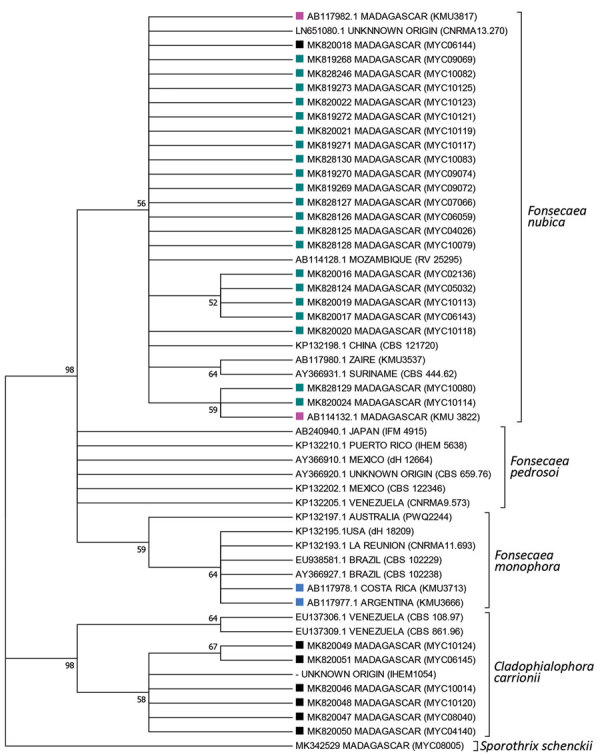
Phylogenetic tree of internal transcribed spacers sequences of fungal isolates from patients with chromoblastomycosis, Madagascar. Tree was constructed by using MEGA7.0 software (https://www.megasoftware.net) and applying the maximum-likelihood method based on the Kimura 2-parameter model (100 bootstrap replicates). Numbers along branches are bootstrap values. GenBank accession numbers are provided. Detailed information for strains is available (Appendix Table). *Sporpthrix schenckii* was used as the outgroup. Dark blue squares, *Fonsecaea nubica* sequences isolated in this study; black squares, *Cladophialophora carrionii* isolated in this study; pink squares, *F. nubica* previously identified as *F. pedrosoi*; light blue squares, *F. monophora* previously identified as *F. pedrosi.*

Comparison of the 31 reliable D1D2 sequences by using the NCBI database identified 20 isolates of *F. pedrosoi*, 4 of *F. monophora*, and 7 of *C. carrionii*. Comparison of the 28 reliable ITS sequences from the International Society of Human and Animal Mycology database confirmed 6 isolates of *C. carrionii* but identified 22 isolates of *F. nubica* as *Fonsecaea* spp. strains (Appendix Table). Identity ranged from 97.8% to 100.0% for *F. nubica* strains and from 99.5% to 99.8% for all *C. carrionii* isolates but 1 (which was 96.3%). Phylogenetic analysis confirmed that 22 *F. nubica* ITS sequences obtained from strains in Madagascar were grouped in the *F. nubica* clade (Figure 3). This clade also includes 2 sequences that correspond to *Fonsecaea* strains collected previously by Institut Pasteur in Madagascar and identified as *F. pedrosoi* (*23*). The *Fonsecaea* strains were isolated from patients who originated from the humid tropical zones of eastern Madagascar, whereas *C. carrionii* was restricted to the southern and eastern regions of this country (Figure 1, panel C).

We used 11 reference strains or isolates identified by ITS sequencing for matrix-assisted laser desorption/ionization time-of-flight (MALDI-TOF) mass spectrometry analysis. We then validated the MSPs by identification of ITS sequenced isolates (Appendix Table). A comparison of the obtained spectra showed that the mean (SD) identification score was 2.01 (0.36) when compared with MSPs already present in reference databases. Statistical tests showed similar identification performance between the 3 culture conditions. In-house MSPs systematically outperformed the 2 sets of MSPs in the Bruker database and the set in the NIH database for *F. nubica* and *C. carrionii* identification, confirming their superiority for discrimination and identification at the species level. Our MSPs identified 1 additional isolate of *F. nubica*, which ITS sequencing failed to identify (MYC10081; Appendix Table).

### Susceptibility of Strains to Antifungal Drugs and Patient Outcomes

A total of 15 *F. nubica* and 5 *C. carrionii* isolates were culturable after thawing for MIC determination. We determined MICs and their geometric means for the 5 antifungal drugs tested (Table 4). MICs were <1 μg/mL for itraconazole and <0.25 μg/mL for posaconazole and isavuconazole for all *F. nubica* and *C. carrionii* isolates. We observed low MICs (<0.031 μg/mL) for terbinafine for all *F. nubica* and 4/5 *C. carrionii* isolates. Amphotericin B appeared to be less active than the other antifungal drugs.

**Table 4:** Minimal inhibitory concentrations of 5 antifungal drugs for *Fonsecaea nubica*) and *Cladophialophora carrionii* (n = 5) strains, Madagascar*

Fungi, drug	MIC, μg/mL and no. isolates										Geometric mean, μg/mL (range)
	0.008	0.015	0.02	0.031	0.062	0.125	0.25	0.5	1.0	2.0	
*F. nubica*, n = 15											
ITZ	0	0	0	5	3	4	1	1	1	0	0.101 (0.031–1.0)
PSZ	0	11	0	2	2	0	0	0	0	0	0.021 (0.015–0.062)
ISZ	1	3	0	10	1	0	0	0	0	0	0.027 (0.008–0.006)
TRB	11	1	2	1	0	0	0	0	0	0	0.012 (0.008–0.003)
AMB	0	0	0	0	1	2	4	6	1	1	0.692 (0.062–4.0)
*C. carrionii*, n = 5											
ITZ	0	0	1	1	0	2	0	1	0	0	0.094 (0.031–1.0)
PSZ	0	0	2	2	0	0	1	0	0	0	0.071 (0.031–0.5)
ISZ	0	0	2	2	0	0	1	0	0	0	0.071 (0.031–0.5)
TRB	2	2	0	0	1	0	0	0	0	2	0.024 (0.008–0.125)
AMB	0	0	0	0	0	0	0	0	5	0	4.595 (2.0–8.0)

*AMB, amphotericin B; ISZ, isavuconazole; ITZ, itraconazole; PSZ, posaconazole; TRB, terbinafine.

A total of 27 (17 with severe cases and 10 with moderate cases) of the 50 chromoblastomycosis patients who were treated (itraconazole, 100 mg 2×/d) could have been followed-up. Patients were treated for 4–26 months independently of disease severity. A complete cure was never achieved, and a major response was seen in only 2 patients with severe forms. Other patients showed only a minor response.

## Discussion

We conducted an epidemiologic study of chromoblastomycosis in Madagascar that went back to 1997, when the studies conducted by Institut Pasteur stopped. Our study confirmed the high endemicity and showed even higher burdens in some regions than described 20 years earlier. Although the climatic–geographic distribution of fungal pathogens was preserved for *Fonsecaea* spp. in the humid tropical climate of the east, north, and northwest regions and *C. carrionii* in the semiarid climate in the southern region, molecular identification led to a revision of *F. nubica* previously described as *F. pedrosoi* (*9*,*24*,*25*). On the basis of this study, we were able to develop and routinely implement molecular analyses in Madagascar, making positive species identification possible.

We report high regional prevalences of 1.47 cases/100,00 persons for the Sava region and 0.8 cases/100,000 persons for the Anosy region. These 2 regions were already perceived to be the major foci of the disease. These prevalences exceed the prevalence of 0.5 cases/100,000 persons estimated 20 years ago (*9*). Other regions, such as Amoron’i Mania, Melaky, or Vatovavy Fitovinany, had lower prevalences (0.31–0.38 cases/100,000 persons), similar to prevalences previously described (*9*). The frequency of chromoblastomycosis in some areas of western and southwestern Madagascar are unknown because these areas were not investigated by advanced consultation campaigns. More recently, Queiroz-Telles reported a prevalence of 0.26 cases/100,000 persons in Madagascar (*26*). Our results show at least a steady high level of endemicity, suggesting that this country could still be the leading focus of chromoblastomycosis worldwide (*9*). However, a comparison with data reported from other countries is challenging because these data are for mostly cumulative cases or series counts (*1*,*4*,*27**–**29*). Our study also confirms the low prevalence in the central highlands, where climatic conditions are different because of higher altitude (drier and cooler than for the northern and eastern coasts) (*9*).

Use of molecular methods, such ITS sequencing and MALDI-TOF mass spectrometry, enabled us to revise the identification of the *Fonsecaea* species endemic to Madagascar as *F. nubica* instead of *F. pedrosoi*. Phylogenetic analysis grouped all reliable *Fonsecaea* sequences obtained from the strains isolated in Madagascar and 2 sequences previously identified as *F. pedrosoi* (*23*) together into the Nubica clade. In addition, analysis of D1D2 sequences showed that the NCBI database was not reliable for identification of *F. nubica* at the species level.

The climato–geographic locations for case-patients infected with *F. nubica* corresponded to those described for *F. perdosoi* in the humid northern and eastern tropical coasts (*1**,**9*). Concerning the second causative pathogen (*C. carrionii*) found in Madagascar, we confirmed its location in the arid southern part of Madagascar. However, we detected 3/7 cases in patients who did not report any trip to the southern region and who originated from the humid zones (*1**,**9*). These results might suggest a larger distribution and lower restrictive climatic conditions for this species.

Patients with chromoblastomycosis were mostly men and farmers, and lesions were located mostly on lower limbs. Most patients were involved in raising crops, working with bare hands and feet, animal husbandry, rearing of livestock (e.g., pigs or zebu cattle) near plantations, and woodcutting and charcoal-producing activities. In the Sava region, in which we found the highest prevalence, vanilla, coffee, sugar cane, and pineapple production are the main activities. In the dry southern area, the second focus of chromoblastomycosis, the flora are characterized by forests of thorny plants of the family *Didieraceae* and euphorbias. This region also contains sisal, which is used for manufacture of rope; eucalyptus, which is used for charcoal production; and cacti, which are used for construction materials (*2*). These rural activities provide many risks for injury by thorny plants or cutting leaves for persons working with bare hands and feet.

Clinical manifestations were mostly polymorphous, with an association of plaques and nodular and warty tumorous features, characterized by pink pimples with a typical cauliflower appearance. Nearly half of the patients were not available for follow-up examinations, mostly because of remoteness of their homes, lack of public transport in rural areas, and disabilities caused by their extensive lesions. We confirmed that chromoblastomycosis lesions are mostly refractory because only 7.0% of the patients showed major improvement. Nevertheless, observance of the treatment and monitoring of therapeutic drug use could not be assessed. Thus, we do not know if the lack of cure was caused by ineffectiveness of itraconazole or low observance of use or low absorption of this drug.

In conclusion, after 20 years without data, our study and update of the epidemiology of chromoblastomycosis in Madagascar confirms its high endemicity. This disease shows a high prevalence in the 2 main disease loci of 1.47 cases/100,00 persons in the northeast region and 0.8 cases/100,000 persons the southeast region of this country. Chromoblastomycosis has persisted in Madagascar, and its burden might be even greater than before, which fully supports the recognition by the World Health Organization that chromoblastomycosis is a neglected tropical disease. On the basis of this recent international effort, national control programs should be conducted to ensure prevention, improve management through the earlier detection of lesions, and facilitate access to treatment.

**Appendix.** Additional information on endemic chromoblastomycosis caused predominantly by *Fonsecaea nubica*, Madagascar.
