# Supplementary material for: Endemic Chromoblastomycosis Caused Predominantly by Fonsecaea nubica, Madagascar
Source: Emerg Infect Dis. 2020 Jun;26(6):1201–11. doi: 10.3201/eid2606.191498 (PMC7258462; doi:10.3201/eid2606.191498)
Supplement: Supplementary file 1 — Appendix. Additional information on endemic chromoblastomycosis caused predominantly by Fonsecaea nubica, Madagascar. [file 19-1498-Techapp-s1.pdf]

Article DOI: https://doi.org/10.3201/eid2606.191498

Endemic Chromoblastomycosis Caused Predominantly by *Fonsecaea nubica*, Madagascar

Appendix

Appendix Table. Characteristics of clinical isolates from patients with suspected chromoblastomycosis in Madagascar and reference strains used in this study*

Code	Species	GenBank accession no. for D1D2, ITS	Source	Origin	Panfugal PCR for S/C		Specific PCR for S/C		MALDI-ToF	
					NL-1/NL-4	ITS1/ITS4	Ccar-F/Ccar-R	Fon-F/Fon-R	MSP	ID
MYC04002	Unidentified	NA, NA	Biopsy	Vatovavy Fitovinany	N/P	P/N	N/N	N/NA	NA	NA
MYC04004	*Fonsecaea* sp.	NA, NA	Biopsy	Vatovavy Fitovinany	P/P	P/NA	N/NA	N/P	NA	NA
MYC08006	*Fonsecaea* sp.	MK828325, NA	Biopsy	Analanjirofo	P/P	P/NA	N/N	N/P	NA	NA
MYC10010	*Fonsecaea* sp.	NA, NA	Biopsy	Melaky	P/P	P/NA	N/NA	P/P	NA	NA
MYC10011	*Fonsecaea* sp.	NA, NA	Squama	Vatovavy Fitovinany	P/P	P/NA	N/NA	P/P	NA	NA
MYC10014	*Cladophialophora carrionii*	MK828362, MK820046	Squama	Androy	P/P	P/P	N/P	N/N	X	NA
MYC03022	Unidentified	NA, NA	Biopsy	Sofia	NA/NA	NA/NA	N/NA	NA/NA	NA	NA
MYC04026	*Fonsecaea nubica*	MK828326, MK828125	Biopsy	Alaotra Mangoro	N/P	N/P	NA/N	NA/P	NA	X
MYC05032	*F. nubica*	MK828327, MK828124	Squama	Amoron I Mania	N/P	N/P	NA/N	NA/P	NA	NA
MYC06033	Unidentified	NA, NA	Biopsy	Androy	N/P	N/P	NA/N	NA/N	NA	NA
MYC08040	*C. carrionii*	MK828363, MK820047	Biopsy	Atsimo Andrefana	N/P	N/P	NA/P	NA/N	NA	NA
MYC09041	Unidentified	NA, NA	Biopsy	Analamanga	NA/NA	NA/NA	NA/NA	NA	NA	NA
MYC03048	Unidentified	NA, NA	Biopsy	Vakinankaratra	N/P	N/N	N/N	N/N	NA	NA
MYC06057	Unidentified	NA, NA	Biopsy	Amoron I Mania	P/P	N/N	N/N	N/N	NA	NA
MYC06059	*F. nubica*	MK828328, MK828126	Biopsy	Sofia	P/P	P/P	N/N	N/N	NA	NA
MYC07066	*F. nubica*	MK828329, MK828127	Biopsy	Vatovavy Fitovinany	P/P	N/P	N/NA	N/P	NA	NA
MYC09069	*F. nubica*	MK828330, MK819268	Biopsy	Sava	P/P	N/P	N/N	P/P	NA	X
MYC09072	*F. nubica*	MK828331, MK819269	Biopsy	Alaotra Mangoro	P/P	N/P	N/N	N/P	NA	X
MYC09074	*F. nubica*	MK828332, MK819270	Biopsy	Analamanga	P/P	N/P	N/N	N/P	NA	NA
MYC10079	*F. nubica*	MK828333, MK828128	Biopsy	Vatovavy Fitovinany	P/P	N/P	N/N	N/P	NA	NA
MYC10080	*F. nubica*	MK828334, MK828129	Biopsy	Sava	P/P	N/P	N/N	N/P	NA	X
MYC10081	*F. nubica*	NA, NA	Biopsy	Sava	P/P	N/P	N/N	N/P	NA	X
MYC10082	*F. nubica*	MK828336, MK828246	Biopsy	Sava	P/P	N/P	N/N	N/P	NA	X
MYC10083	*F. nubica*	MK828337, MK828130	Biopsy	Sava	P/P	N/P	N/N	N/P	X	NA
MYC04100	*Fonsecaea* sp.	MK828338, NA	Biopsy	Ihorombe	N	N/N	N/N	N/P	NA	NA
MYC05103	Unidentified	NA, NA	Biopsy	Atsinanana	N	N/N	N/N	N/N	NA	NA
MYC08112	Unidentified	NA, NA	Biopsy	Alaotra Mangoro	P/P	N/N	N/N	N/N	NA	NA
MYC10113	*F. nubica*	MK828339, MK820019	Biopsy	Sava	P/P	N/P	N/N	P/P	NA	NA
MYC10114	*F. nubica*	MK828340, MK820024	Biopsy	Sava	P/P	N/P	N/N	P/P	NA	NA
MYC10115	Unidentified	NA, NA	Biopsy	Sava	P/P	N/P	N/N	N/N	NA	NA
MYC10116	Unidentified	NA, NA	Biopsy	Sava	P/NA	N/NA	N/NA	P/NA	NA	NA
MYC10117	*F. nubica*	MK828341, MK819271	Biopsy	Sava	P/P	N/P	N/N	P/P	X	NA

Code	Species	GenBank accession no. for D1D2, ITS	Source	Origin	Panfugal PCR for S/C		Specific PCR for S/C		MALDI-ToF	
					NL-1/NL-4	ITS1/ITS4	Ccar-F/Ccar-R	Fon-F/Fon-R	MSP	ID
MYC10118	*F. nubica*	MK828342, MK820020	Biopsy	Sava	P/P	N/P	N/N	P/P	NA	NA
MYC10119	*F. nubica*	MK828343, MK820021	Biopsy	Sava	P/P	N/P	N/N	P/P	NA	NA
MYC10120	*C. carrionii*	MK828364, MK820048	Biopsy	Analanjirofo	P/P	N/P	N/P	0/N	NA	NA
MYC10121	*F. nubica*	MK828344, MK819272	Biopsy	Sava	P/P	N/P	N/N	P/P	NA	X
MYC10122	*Fonsecaea* sp.	MK828345, NA	Biopsy	Sava	P/P	N/P	N/N	P/P	NA	NA
MYC10123	*F. nubica*	MK828346, MK820022	Biopsy	Sava	P/P	N/P	N/N	P/P	NA	X
MYC10124	*C. carrionii*	NA, MK820049	Biopsy	Vatovavy Fitovinany	P/P	N/P	N/P	N/N	NA	X
MYC10125	*F. nubica*	MK828347, MK819273	Biopsy	Alaotra Mangoro	N/P	N/P	N/N	P/P	NA	X
MYC12128	Unidentified	NA, NA	Biopsy	Haute Matsiatra	N/N	N/N	N/N	N/N	NA	NA
MYC01133	Unidentified	NA, NA	Biopsy	Analamanga	P/NA	P/NA	N/NA	N/NA	NA	NA
MXC02136	*F. nubica*	MK828348, MK820016	Biopsy	Sava	P/P	N/P	N/N	P/P	X	NA
YC04140	*C. carrionii*	MK828365, MK820050	Biopsy	Amoron I Mania	P/P	N/P	N/P	N/N	X	NA
MYC06143	*F. nubica*	MK828349, MK820017	Biopsy	Anosy	P/P	P/P	N/N	P/P	X	X
MYC06144	*F. nubica*	MK828350, MK820018	Biopsy	Anosy	P/P	P/P	N/N	P/P	X	NA
MYC06145	*C. carrionii*	MK828366, MK820051	Biopsy	Anosy	P/P	N/P	N/P	N/NA	NA	X
MYC06146	*C. carrionii*	MK828367, NA	Biopsy	Anosy	P/P	N/P	N/N	N/N	NA	NA
MYC06148	Unidentified	NA, NA	Biopsy	Anosy	P/P	P/N	N/NA	N/NA	NA	NA
MYC06149	Unidentified	NA, NA	Biopsy	Anosy	P/P	P/P	N/NA	N/NA	NA	NA
MYC08005	*Sporothrix schenckii*	MK249805, MK342529	Biopsy	Analamanga	P/P	N/P	N/N	N/N	NA	NA
CBS 121720	*F. nubica*	NA, KP132198	Clinical strain	China	NA/P	NA/P	NA/N	NA/P	NA	X
CBS 444.62	*F. nubica*	NA, AY366931.1	Clinical strain	Surinam	NA/P	NA/P	NA/N	NA/P	X	NA
IHEM1054	*C. carrionii*	NA, NA	NA	NA	NA/P	NA/P	NA/P	NA/N	X	NA
CBS 269.37	*F. monophora*	NA, AY857511.1	Clinical strain	Brazil	NA/P	NA/P	NA/N	NA/P	X	NA
IHEM 5638	*F. pedrosoi*	NA, NA	Clinical strain	Puerto Rico	NA/P	NA/P	NA/N	NA/P	X	NA

*C, colony; IHEM, Mycology Laboratory of the Institute of Hygiene and Epidemiology; ITS, internal transcribed spacer; MALDI-ToF, matrix-assisted laser desorption/ionization time-of-flight mass spectrometry; MSP, main spectrum profile; N, negative; NA, not available; P, positive; S, sample, X, strain used either for MSP creation or identified with an in-house MSP.
